# Primary Intracranial Meningeal Melanocytoma with Malignant Transformation: A Case Report and Comparison of Early Versus Late Immunotherapy Interventions

**DOI:** 10.3390/curroncol32110595

**Published:** 2025-10-24

**Authors:** Yi-Qi Zhang, Kun-Ming Rau, Cheng-Loong Liang, Yu-Duan Tsai, He-Tai Jheng, Kuo-Wei Wang

**Affiliations:** 1 Department of Neurosurgery, E-Da Hospital, Kaohsiung 824005, Taiwan; ed114109@edah.org.tw (Y.-Q.Z.);; 2 Department of Oncology, E-Da Cancer Hospital, Kaohsiung 824005, Taiwan; 3 Department of Neurosurgery, E-Da Cancer Hospital, Kaohsiung 824005, Taiwan

**Keywords:** immune checkpoint inhibitors, melanocytoma, malignant transformation, pembrolizumab, ipilimumab, nivolumab

## Abstract

Primary meningeal melanocytoma is an uncommon, pigmented neoplasm that rarely undergoes malignant transformation, and therapeutic guidelines remain undefined. We report a 43-year-old woman who initially presented with a sudden headache and a right temporal intraparenchymal mass. Subtotal resection revealed a melanocytoma (WHO grade I); residual tumor was treated with Gamma Knife. About 15 months later, she deteriorated rapidly due to malignant transformation with cerebral hemorrhage and spinal leptomeningeal metastasis. Pembrolizumab was initiated within four weeks of the malignant diagnosis and produced transient neurological improvement. Due to symptomatic progression, ipilimumab plus nivolumab was commenced and achieved temporary radiographic stabilization, but the patient succumbed to diffuse progression later. Including this case, only five intracranial melanocytomas with malignant transformation treated with immune checkpoint inhibitors have been reported. Our experience supports initiating immunotherapy promptly after malignant transformation and suggests that sequential dual-agent blockade may modestly extend disease control.

## Simple Summary

Primary meningeal melanocytoma is a rare tumor that can occasionally transform into an aggressive melanoma, but there are no clear treatment guidelines. We describe a woman who initially had a benign melanocytoma that later had malignant transformation with multiple organ metastasis. The patient received immunotherapy early after malignant transformation; the response was temporary. By reviewing all similar cases, we found that early initiation of immunotherapy—within one month of malignant transformation—was associated with better short-term neurological and functional outcomes, while delayed or absent therapy offered little benefit. This case underscores the importance of recognizing malignant transformation promptly and considering early immunotherapy, even in rapidly proliferating tumors, to improve disease control.

## 1. Introduction

Primary meningeal melanocytoma is a rare, pigmented neoplasm that arises from leptomeningeal melanocytes, with an incidence of approximately 1 in 10 million cases per year. These neoplasms are generally benign (World Health Organization Grade I) but have a high local recurrence rate (~46% five years after gross total resection) [1]. The malignant transformation of a melanocytoma into an aggressive meningeal melanoma is uncommon, with only a handful of well-documented cases reported [2]. However, when malignancy occurs, the clinical course often accelerates with diffuse leptomeningeal dissemination and a historically poor prognosis despite multimodal therapy [1,3].

Until recently, treatment options for malignant central nervous system melanocytic tumors were limited to surgical resection, radiation, and chemotherapy (e.g., temozolomide), which minimally affected survival [1,3]. Immune checkpoint inhibitors have revolutionized the management of metastatic melanoma, significantly improving patient outcomes [2]. However, the effects of early (within one month of malignant transformation) versus late (more than six months after transformation) immunotherapy intervention on malignantly transformed melanocytoma remain unclear.

Herein, we present a case of intracranial melanocytoma with malignant transformation and metastasis managed with early immunotherapy and compare early versus late immunotherapy interventions in this rare scenario.

## 2. Detailed Case Description

**Figure 1:**
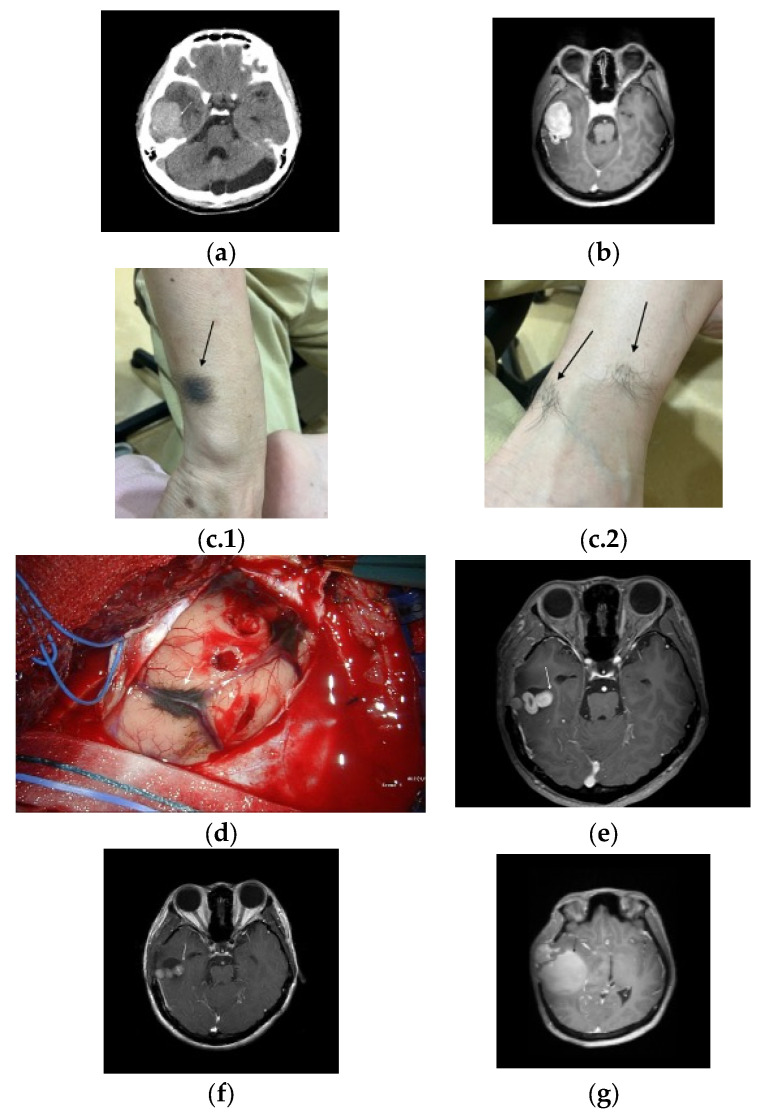
(**a**) Brain computed tomography with contrast shows a mass (arrow) in the right temporal lobe accompanied by perifocal edema, along with an incidental arachnoid cyst identified in the cerebellar region. (**b**) Brain magnetic resonance imaging with contrast shows a well-defined, intensely and homogeneously enhanced mass lesion (arrow) in the right temporal lobe. (**c.1**,**c.2**) Multiple pigmented skin lesions (arrows).(**d**) Intraoperative photograph shows a hypervascular black-colored tumor (arrow) and marked adhesions in the temporal lobe and petrosal vein. (**e**) MRI with contrast (two-months postoperative follow-up) showed small residual tumor (arrow). (**f**) MRI with contrast (three months post-gamma knife radiosurgery) shows residual tumor (arrow). (**g**) MRI with contrast showed tumor recurrence with cystic changes (arrow).

A 43-year-old woman presented to the emergency department after a seizure and consciousness disturbance. Brain computed tomography (CT) upon admission revealed a right temporal lobe mass with perifocal edema and an incidental arachnoid cyst in the cerebellum (Figure 1a). Subsequent magnetic resonance imaging (MRI) confirmed a right temporal lobe mass with intense homogeneous enhancement suggestive of a meningioma (Figure 1b). A physical examination revealed several pigmented cutaneous lesions (Figure 1c.1,c.2).

The patient underwent temporal craniotomy, during which a hypervascular black-colored tumor was partially excised because of adhesions to the temporal lobe and petrosal vein (Figure 1d). The pathological analysis revealed a melanocytoma supported by a well-circumscribed architecture, bland cytology with very low mitotic activity (<1/10 HPF), a low Ki-67 proliferative index, absence of necrosis, and lack of diffuse PRAME expression. The patient was discharged in a stable condition.

Two months later, a follow-up MRI revealed a residual tumor (Figure 1e), and gamma knife radiosurgery was performed. However, a subsequent surveillance MRI revealed a small but persistent residual tumor burden (Figure 1f).

Approximately 15 months after the initial diagnosis, the patient experienced acute vertigo and severe headaches. Emergency CT revealed an intracerebral hemorrhage within the tumor, which caused a significant midline shift (Figure 1g). An emergency craniectomy was performed, and the pathological examination revealed malignant melanoma transformation characterized by a high proliferative index (Ki-67: 50%) and diffuse nuclear PRAME positivity with concordant strong SOX10 and HMB-45 expression (and only weak/focal S100). Because National Health Insurance does not cover NGS or extended molecular panels and the patient had limited resources, comprehensive genomic testing (e.g., *TERT* promoter, *CDKN2A*/p16, BAP1) was not pursued.

Postoperatively, a systemic evaluation, including positron emission tomography, was conducted to rule out the presence of extracranial primary malignancies. An excisional biopsy of the pigmented lesions on the left foot, right flank, and right buttock revealed intradermal nevi (Figure 1c.1,c.2), which excluded these as primary tumor sources.

**Figure 2:**
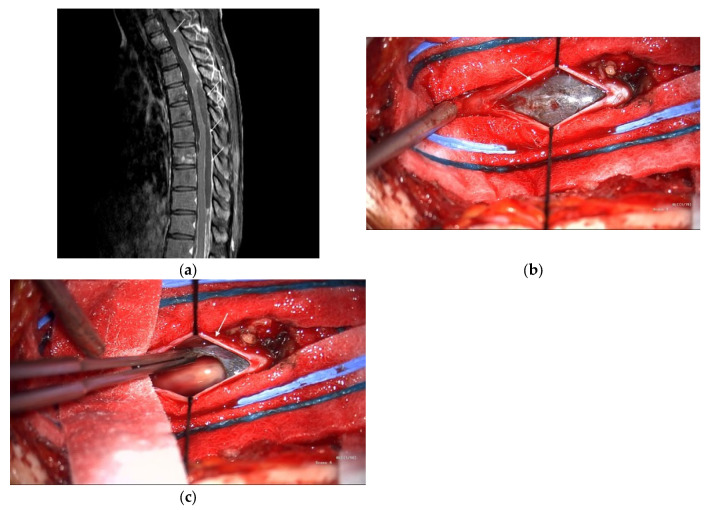
(**a**) Spine magnetic resonance imaging (MRI) with contrast shows increased enhancement (arrows) along the dural sac of the thoracolumbar spine with several nodular enhancements and increased enhancement of the nerve roots of the lumbar spine. (**b**) Intraoperative photography showed subdural tumors (arrow). (**c**) Intraoperative photography showed tumor excision.

Subsequently, the patient developed acute paraplegia. MRI revealed a vertebral metastasis and leptomeningeal dissemination. Owing to extensive metastases and poor surgical candidates, the patient was referred to the Medical Oncology Department and began pembrolizumab therapy. Single-agent pembrolizumab was selected because of rapid clinical deterioration, concerns about the toxicity of upfront dual-agent therapy, and cost constraints. After nine immunotherapy cycles, the patient’s muscle strength improved, and they regained the ability to walk. However, widespread metastatic disease involving the vertebrae and spinal leptomeninges remained visible on MRI (Figure 2a); the liver, mesentery, pelvis, and multiple bone sites were also involved. Thoracic laminectomy was performed to debulk the spinal tumor; however, only the dorsal component could be removed because the spinal cord was adhered to the ventral tumor (Figure 2b,c). The patient’s postoperative course was uneventful, and they returned to the oncology department for further treatment.

**Figure 3:**
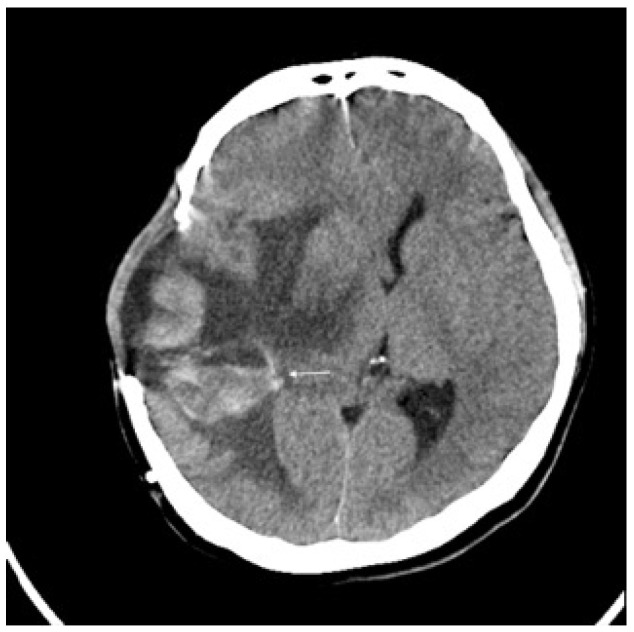
Brain CT without contrast showed recurrent tumor (arrow) with acute hemorrhage.

**Figure 4:**
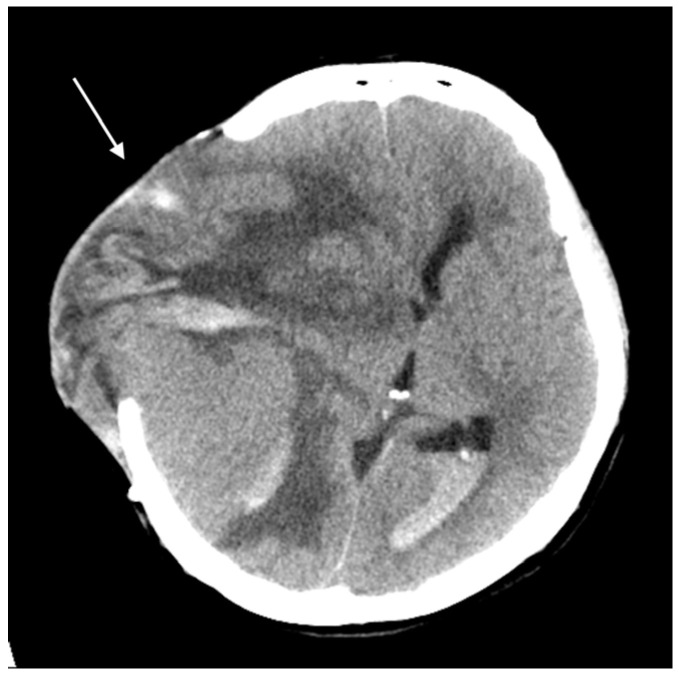
Brain CT without contrast showed a huge recurrent tumor with hemorrhage and cerebral edema (arrow).

Three months later, the patient experienced rapid clinical deterioration due to a recurrent hemorrhagic tumor in the right temporal lobe (Figure 3). Repeat craniectomies with partial tumor and hematoma evacuation were performed (Figure 4). During the subsequent hospitalization, the patient underwent four additional craniectomies for recurrent tumor bleeding. The patient died one month after the final craniectomy.

## 3. Discussion

Recent case series and reports have examined management strategies and outcomes for primary meningeal melanocytic tumors [1,2,3,4]. Gross-total resection remains the cornerstone of therapy; however, long-term follow-up shows that relapse and, in some cases, malignant transformation can occur even after apparently complete excision [4]. These observations indicate that current modalities do not reliably prevent recurrence and that disease-specific mortality remains a concern, supporting a comparison of our patient’s management with the existing literature [1,2,3,4].Particularly the potential benefits of early systemic therapies (e.g., immune checkpoint inhibitors).

Including our patient, only five cases of intracranial meningeal melanocytoma with confirmed malignant transformation have been reported (Table 1), Refs. [1,3,4,5] comprising male and female patients aged 37 to 71 years. The primary tumor location was intracranial (temporal/petroclival region, cerebellopontine angle, and frontal lobe). In all cases, the initial tumor was pathologically benign or intermediate-grade melanocytic but eventually underwent malignant progression with either local invasive recurrence or metastatic spread to the leptomeninges, brain, or systemic organs. Notably, the time to malignant transformation (latency) varied widely, ranging from virtually immediate (simultaneous melanocytoma and melanoma at diagnosis) to over a decade.

The survival duration after malignant transformation is relatively similar among all cases, ranging from ~4 to 18 months post-transformation [1,2,4,5]. However, the malignant potential of these cases varied considerably based on the Ki-67 proliferation index, a well-recognized biomarker of cellular proliferation and aggressiveness in tumors. Our patient had a Ki-67 index of 50%, which was distinctly higher than those previously reported. High Ki-67 index values typically correlate with more aggressive clinical behavior and a poorer prognosis [6,7]. Therefore, our patient’s extremely high Ki-67 value theoretically predicted rapid clinical deterioration and shorter overall survival compared with the other cases.

Despite this, our patient experienced a similar, if not slightly better, survival duration (~15 months after the malignant transformation; ~30 months after the initial diagnosis) compared to the other cases with lower Ki-67 indices. For instance, the patients in the reports by Roser et al. [1] and Koch et al. [3] rapidly deteriorated within 4–5 months post-malignancy without immunotherapy, despite comparatively lower Ki-67 values (25% and unknown, respectively). This contrasts with our patient’s experience, strongly suggesting a clinical benefit of initiating immunotherapy early. Our patient received pembrolizumab within approximately one month of receiving a malignant diagnosis.

The patient described by Küsters et al. [5] also had a notably lower Ki-67 index (10%) but received delayed immunotherapy; ipilimumab was initiated approximately seven months after metastasis detection. Despite the lower malignant potential and eventual administration of immunotherapy, the patient’s intracranial disease progressed, and no significant clinical improvement was noted. Their survival duration was also similar to that of our patient, who had a markedly higher malignant potential. Gempt et al. [4] described a patient with a Ki-67 index of 12% who survived for ~18 months after the initial malignant diagnosis using only surgery and radiation therapy without immunotherapy. Thus, even in relatively lower-grade malignant cases, delayed or absent immunotherapy does not guarantee improved outcomes.

Notably, our patient is the only one to sequentially receive two distinct immune checkpoint inhibitors (initially pembrolizumab and later ipilimumab upon disease progression). Clinically, we observed meaningful, although temporary, symptomatic improvements with pembrolizumab, including ambulatory function recovery. Although eventual disease progression occurred, the use of dual immunotherapy, even when administered sequentially, potentially contributed to an extended survival period comparable to that of patients with considerably lower Ki-67 indices.

Diagnosing primary meningeal melanocytic tumors remains challenging because the spectrum from melanocytoma to melanoma shows overlapping histological and immunohistochemical features [8]. Our case was initially classified as a melanocytoma based on bland histology and low mitotic index; subsequent recurrences showed increased mitotic activity and cytological atypia consistent with malignant melanoma. Immunohistochemistry was crucial for confirming the melanocytic origin, with diffuse positivity for S100, SOX10, HMB45 and Melan-A and a Ki-67 index increasing from <1% to 5–10% across resections. However, Ki-67 alone is not sufficient to distinguish benign from malignant lesions. Ki-67 values often rise during malignant transformation; reports show that the Ki-67 labeling index is usually below 3% in melanocytoma but increases in intermediate-grade or recurrent tumors [9].

Recent reviews highlight that molecular profiling can detect *GNAQ*, *GNA11*, *SF3B1*, *EIF1AX* and *BAP1* mutations in circumscribed meningeal melanocytic tumors, whereas *NRAS* and *BRAF* mutations are more typical of diffuse tumors [10]. Loss of *CDKN2A* (encoding p16^INK4A) has been shown to cooperate with *NRAS* mutations to induce melanoma and may play a role in malignant progression [11]. Immunohistochemical markers such as PRAME and p16 may further aid classification; PRAME is highly specific for melanoma and is uniformly negative in benign nodal nevi but positive (>50% tumor cells) in metastatic melanoma [12,13]. In our institution, next-generation sequencing and extended molecular panels are not routinely covered by the national health system, and patients must pay for these tests out of pocket. Given the patient’s limited financial resources, comprehensive genomic testing—including assessment for *TERT* promoter, *CDKN2A*/p16, *BAP1* and other mutations—was not performed, and tissue reserves were prioritized for diagnostic immunohistochemistry. PRAME staining was performed but showed focal rather than diffuse expression, while *BAP-1* and p16 immunostains could not be obtained due to both tissue limitations and cost constraints. Future cases would benefit from comprehensive molecular testing, including NGS panels for *GNAQ*/*GNA11*, *BRAF*/*NRAS*, *TERT* promoter and tumor suppressor genes (e.g., *BAP1*), as well as immunohistochemistry for p16 and PRAME, to better delineate the tumor’s origin and to guide prognosis and targeted therapy [14].

Advances in targeted therapy and immune checkpoint blockade have begun to reshape the therapeutic landscape for central nervous system melanocytic tumors. Experience from melanoma brain metastases shows that combining *BRAF*/MEK inhibitors with PD-1/CTLA-4 checkpoint inhibitors improves intracranial tumor control [15], and some authors have extrapolated this approach to primary meningeal melanoma.

A recent review of *GNAQ*/*GNA11*-mutated tumors emphasized that MEK inhibitors alone have limited efficacy but may yield better results when combined with immune checkpoint blockade [16]. Case reports also suggest that *SERPINB3/4* mutations correlate with responses to PD-1 or CTLA-4 inhibition [17]. Together, these data suggest that genetic analysis (e.g., *BRAF*, *GNAQ*/*GNA11*, *SERPINB3/4*) and the judicious use of targeted agents and checkpoint inhibitors should be considered when discussing treatment options for patients with malignant transformation or recurrence.

Moreover, reports on leptomeningeal disease indicate that intrathecal administration of pembrolizumab or nivolumab is feasible and safe and may prolong survival [18,19]; in an early-phase study of intrathecal nivolumab, the 50 mg dose produced no dose-limiting toxicities and achieved a 26-week survival rate of 44%, supporting further development of intrathecal immunotherapy [19].

There are several limitations to this report. Our report describes only a single patient, which limits the ability to draw broad conclusions about disease behavior or therapeutic efficacy. Comprehensive molecular profiling, including common melanoma-associated mutations in *BRAF*, *NRAS* and the *GNAQ/GNA11* signaling pathway, was not performed in this case. Such mutations have prognostic and therapeutic implications—targeted MEK inhibition or combined MEK/immune-checkpoint blockade may be more effective in tumors with *GNAQ/GNA11* alterations [15,16]—and their absence restricts our capacity to individualize treatment. Finally, although recent case reports and early-phase trials suggest that immune checkpoint inhibitors and intrathecal immunotherapy can be beneficial [18,19], the evidence remains limited to small cohorts, precluding generalization of any observed benefit to all patients with primary meningeal melanocytic tumors.

## 4. Conclusions

In conclusion, our experience strongly supports early immunotherapy interventions for patients with malignant melanocytoma, even in highly aggressive cases characterized by high Ki-67 proliferation indices.

## Figures and Tables

**Table 1:** Clinical characteristics of melanocytoma with malignant transformation treated with early (within one month of confirmed transformation) and late (>6 months after confirmed transformation) immunotherapy.

Citation	Age/ Sex/ Ethnicity	Primary Tumor Location	Metastatic Sites	Treatment Approach	Immunotherapy Timing	Clinical Response	Time to Malignant Transformation	Overall Survival	Ki-67
Present case	43/ F/ Asian	Right temporal (intracranial)	Leptomeningeal (spine); bone, liver, etc.	Surgery (subtotal) + Gamma Knife; Early pembrolizumab; Ipilimumab on progression	Early (~1-month post-transformation)	Improved mobility after pembrolizumab; later decline	~15 months after the initial diagnosis	~15 months PT (2.5 years from initial)	50%
1.	37/ F/ Caucasian	Petroclival (posterior fossa)	Diffuse leptomeningeal spread (brain/spine)	Multiple surgeries; Whole-brain RT; Temozolomide chemotherapy	None ^a^	Rapid progression; no neurological improvement reported	~12 years after identifying the initial tumor	~4 months PT (14 years from initial)	25%
2.	38/ M/ Caucasian	Left CPA	Intracerebral and spinal leptomeningeal seeding	Surgery ×2; Fractionated RT; Temozolomide chemotherapy	None ^a^	Tumor refractory to CRT; neurological decline	~6 years after the initial diagnosis	~5 months PT (6.5 years from initial)	NM
3.	43/ F/ Caucasian	Right parietal (supratentorial)	Liver, pancreas (systemic metastases); recurrent brain lesions	Surgery ×2 (brain); Stereotactic RT/radiosurgery (brain); ALPPS liver resection; Temozolomide; Delayed ipilimumab	Late (~7 months post-metastasis)	Systemic disease stabilized (no new mets) but intracranial progression observed; no motor improvement documented	~4 years after the initial diagnosis	~15 months PT (~63 months from initial)	10%
4.	71/ F/ Caucasian	Right frontal + diffuse melanosis	Diffuse intracranial (leptomeningeal spread)	Surgery (gross total resection); Whole-brain RT	None ^b^	Continued neurological decline (progressive bulbar symptoms)	~0 years (malignant at presentation)	Alive 18 months post-diagnosis ^c^	12%

^a^ Pre-immunotherapy era. ^b^ No systemic therapy. ^c^ The patient has progressive disease but was still alive at the time of manuscript submission. Abbreviations: CPA, cerebellopontine angle; CRT, chemoradiotherapy; F, female; M, male; NM, not mentioned; PT, post-transformation; RT, radiotherapy.

## Data Availability

All data generated or analyzed during this study are included in this published article. Further inquiries can be directed to the corresponding author.
